# Ameliorated chest drain wound closure in patients undergoing uniportal thoracoscopic pulmonary resection

**DOI:** 10.3389/fsurg.2023.1323937

**Published:** 2023-12-19

**Authors:** Ping-Ruey Chou, Chieh-Ni Kao, Yu-Ting Lo, Che-Yu Chuang, Yu-Wei Liu

**Affiliations:** ^1^ Graduate Institute of Medicine, College of Medicine, Kaohsiung Medical University, Kaohsiung, Taiwan; ^2^ Department of General Medicine, Kaohsiung Medical University Hospital, Kaohsiung Medical University, Kaohsiung, Taiwan; ^3^ Department of Surgery, Kaohsiung Medical University Hospital, Kaohsiung Medical University, Kaohsiung, Taiwan

**Keywords:** chest drain, uniportal, VATS (video-assisted thoracic surgery), wound closure, barbed absorbable suture

## Abstract

**Background:**

Although uniportal video-assisted thoracoscopic surgery (VATS) has been performed for a wide array of thoracic diseases, unsightliness and poor wound healing often occur, particularly when a chest drain is placed postoperatively. Different chest drain wound closure (CWC) methods have been introduced with the benefits of cosmesis and patient satisfaction. We aimed to describe our improved CWC technique in this setting and assess its efficacy.

**Methods:**

A total of consecutive 334 patients undergoing uniportal VATS pulmonary resection with single chest drain placement were investigated from 2016 to 2021. The techniques for CWC were classified into the conventional method (35 patients, group A), continuous suture with removal-free stitches (122 patients, group B), and continuous suture with removal-free barbed suture plus topical skin adhesives (177 patients, group C). Perioperative data and complications related to CWC were analyzed.

**Results:**

Group C had a significantly shorter operative time, postoperative hospital stay, and chest tube days than groups A and B (all *p *< 0.01). In terms of chest tube-related complications, there were no statistically significant differences in post-removal pneumothorax, subcutaneous emphysema, incisional effusion leakage, wound dehiscence, or infection. Overall, significant differences in scar scale scores were observed between the groups, where the ameliorated group C was superior to the conventional group A (*p *< 0.01).

**Conclusion:**

The improved CWC technique using continuous sutures with removal-free barbed sutures and topical skin adhesives is simple, safe, and effective. This may be a favorable CWC strategy when performing uniportal VATS, with enhanced patient satisfaction.

## Introduction

Uniportal video-assisted thoracoscopic surgery (VATS) is a minimally invasive surgical technique used to treat lung diseases (1). This approach involves making a single incision through which all surgical instruments, including a camera, are inserted, thereby providing enhanced visualization and precise surgical maneuvers. Compared to multiportal VATS, uniportal VATS offers numerous benefits, including reduced tissue damage to the chest wall, less postoperative wound pain, shorter hospital stays, and cosmesis (2, 3). However, it is important to be aware of the potential chest tube-related complications that occur more frequently in uniportal VATS, such as subcutaneous emphysema and wound infection (4). Because of direct chest tube insertion through a single incision following uniportal VATS, intrathoracic air or fluid leakage into the subcutaneous tissue around the chest tube frequently occurs because of inadequate incision closure using conventional chest drain wound closure (CWC) methods (5). Thus, uniportal VATS often results in poorer incision wound healing and more significant scarring than multiportal VATS (5).

In recent years, modified CWC methods for uniportal VATS have been introduced in terms of suture techniques and materials to improve wound closure and reduce scarring (4–8). Different layers of continuous barbed sutures with removal-free stitches have been demonstrated in previous studies, supporting their feasibility and variety in uniportal VATS settings (4, 7, 8). Only a few studies have compared the clinical outcomes of the modified and conventional CWC methods in patients undergoing uniportal VATS (6, 7). Similarly, our surgical team has developed different CWC methods for uniportal VATS over the past six years. In this study, we compared our improved CWC technique with the conventional method in terms of feasibility, effectiveness, and complications following uniportal VATS pulmonary resection.

## Material and methods

### Patients

This retrospective cohort study was conducted between August 2016 and July 2021 at a single institute by a single surgical team in Southern Taiwan. The Institutional Review Board of Kaohsiung Medical University Hospital approved this study and waived the requirement for written informed consent [KMUHIRB-E(II)-20220192].

**Figure 1:**
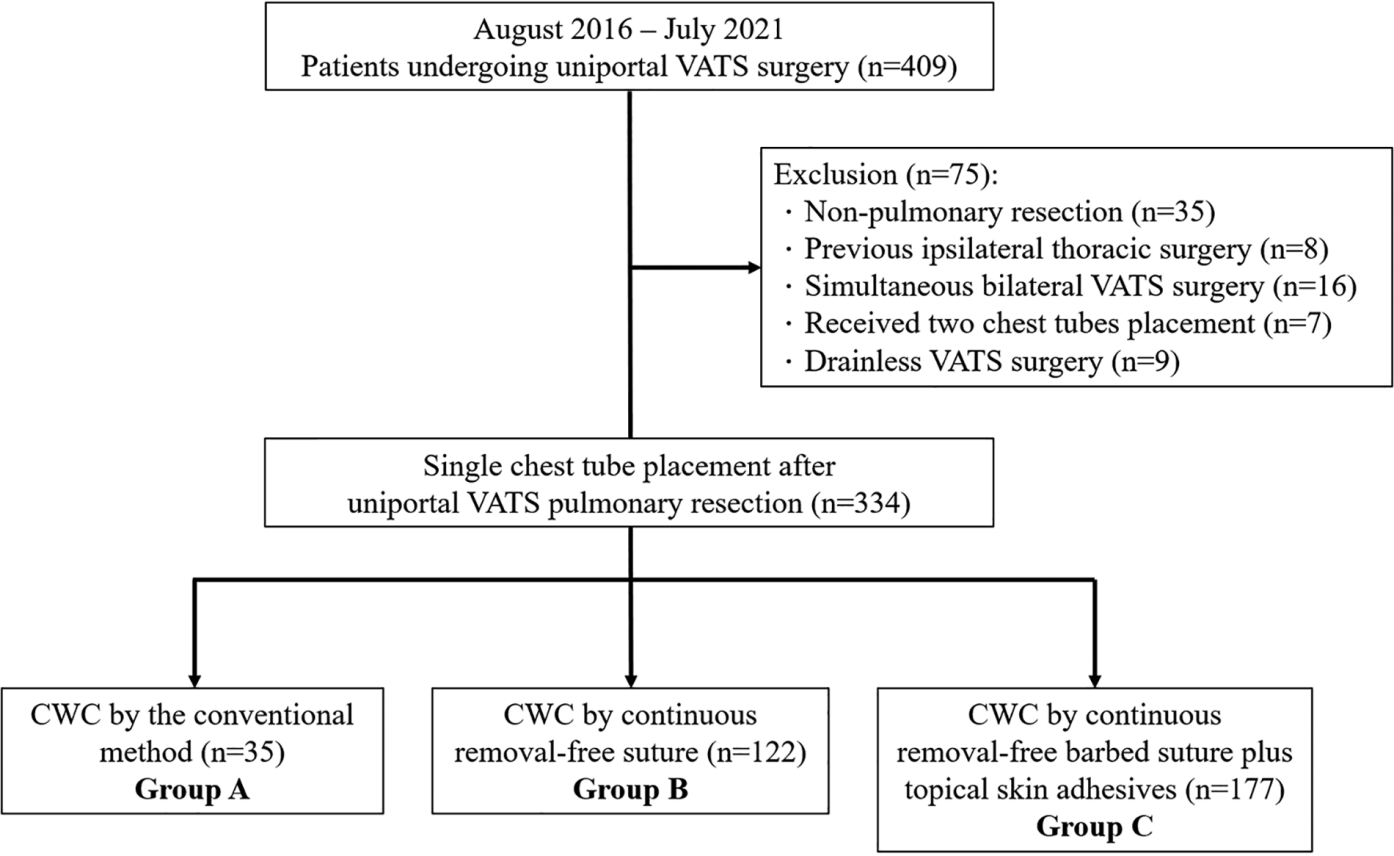
Flow diagram nof patient recruitment. VATS: video-assisted thoracoscopic surgery, CWC, chest drain wound closure.

Four hundred and nine patients undergoing uniportal VATS procedures were evaluated. We excluded patients who underwent non-pulmonary resection (*n* = 35), those with a previous ipsilateral thoracic surgery (*n* = 8), those who underwent surgery for simultaneous bilateral lung resection (*n* = 16), those who had two chest tubes (*n* = 7), or no chest drain placement (*n* = 9), resulting in 334 patients with complete analysis of the CWC method after uniportal VATS resection for lung neoplasms. With time and accumulated experience performing uniportal VATS, the CWC method has evolved and been modified. The patients were divided into three groups: (1) 35 patients receiving the conventional method (group A), (2) 122 patients who received sutures with the removal-free stitch (group B), and (3) 177 patients who received continuous sutures with removal-free barbed sutures plus topical skin adhesives (group C, namely, the ameliorated group) (Figure 1). Patient data, including demographic characteristics, perioperative data, and chest drain-related outcomes, were collected from the electronic medical records.

### Suture material and technique

**Figure 2:**
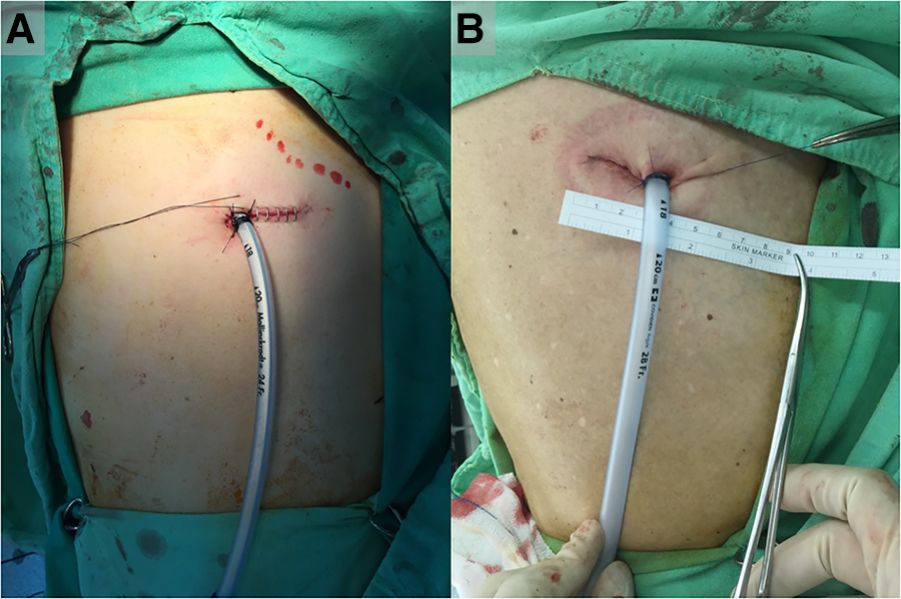
Chest drain wound closure methods among groups A and B. (**A**) In group A, an anchoring suture with 3-0 Nylon was placed to fix the drain, and another suture was left to seal the incision after the chest tube was withdrawn. (**B**) In group B, continuous intradermal suture using 3-0 Monocryl was performed, and the chest drain was bypassed. The last suture was inserted 1 cm from the lowest edge of the skin incision with a 3–5 cm thread left outside the skin. A 3-0 Nylon suture was used to anchor the drain.

Group A: In the conventional method, following appropriate placement of the chest drain under thoracoscopic guidance, the interrupted suture technique with 2-0 Vicryl and a skin stapler (Appose™, Covidien, Minneapolis, MN, USA) was used to close the muscle, subcutaneous tissue, and skin of the chest drain wound after uniportal VATS. An anchoring suture with 3-0 Nylon was placed to fix the drain, and another suture was left to seal the incision after the chest tube was withdrawn (Figure 2A). The tube was removed after the patients maintained a Valsalva maneuver at the end of full inspiration, while the adjacent tissue was pinched tightly, and the wound was closed immediately with the thread left in advance by tying three knots. At last, Vaseline*®* Petrolatum gauze was used to apply pressure the incision after the chest drain was removed.

In group B, the chest drain was placed directly into the incision as usual, close to the lower edge of the incision. Intermittent suturing with 2-0 Vicryl was performed on the muscle and subcutaneous layers from the uppermost to the lowest edge of the incision. Continuous intradermal suture using 3-0 Monocryl was performed, and the chest drain was bypassed. The last suture was inserted 1 cm from the lowest edge of the skin incision with a 3–5 cm thread left outside the skin. A 3-0 Nylon suture was used to anchor the drain (Figure 2B). When the chest drain was ready to be removed, the anchoring suture was cut off and the drain was withdrawn in a manner similar to that in group A. The secured thread was pulled forward to tighten the sutures. Wound dressing was performed according to the aforementioned steps in group A (Supplementary Video S1).

**Figure 3:**
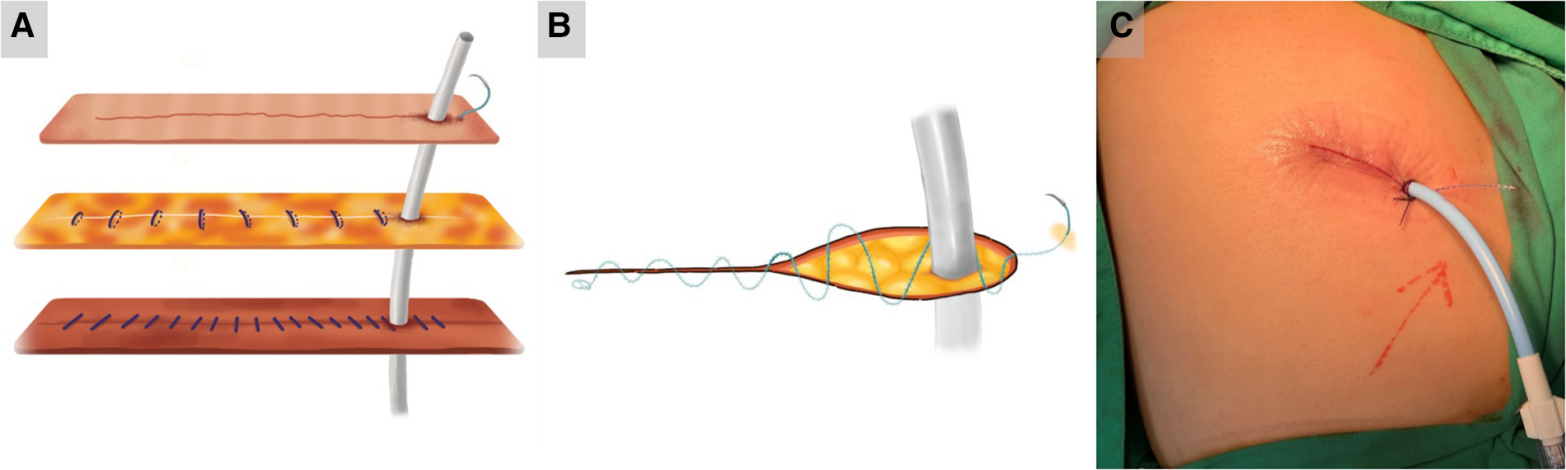
The improved chest drain wound closure method in group C. (**A**) In group C, intermittent suturing with 2-0 Vicryl was performed on the muscle and subcutaneous layer from the uppermost to the lowest edge of the incision. (**B**) A continuous intradermal suture using a unidirectional absorbable 3-0 V-loc (Covidien, Minneapolis, MN, USA) barbed suture was performed in the same manner after bypassing the chest drain. (**C**) The last suture was inserted 1 cm away from the lowest edge of the skin incision with a 3–5 cm thread left outside the skin.

In group C, the chest drain was placed directly into the incision as usual, close to the lower edge of the incision. For our modified technique, intermittent suturing with 2-0 Vicryl was performed on the muscle and subcutaneous layer from the uppermost to the lowest edge of the incision (Figure 3A). In contrast, a continuous intradermal suture using a unidirectional absorbable 3-0 V-loc (Covidien, Minneapolis, MN, USA) barbed suture was performed in the same manner after bypassing the chest drain (Figure 3B). The last suture was inserted 1 cm away from the lowest edge of the skin incision with a 3–5 cm thread left outside the skin (Figure 3C and Supplementary Video S2*)*. After removal of the chest drain, As described above, topical skin adhesives (SURGISEAL®) was immediately applied to cover the wound (Supplementary Video S3).

### Assessment items and follow-up

**Figure 4:**
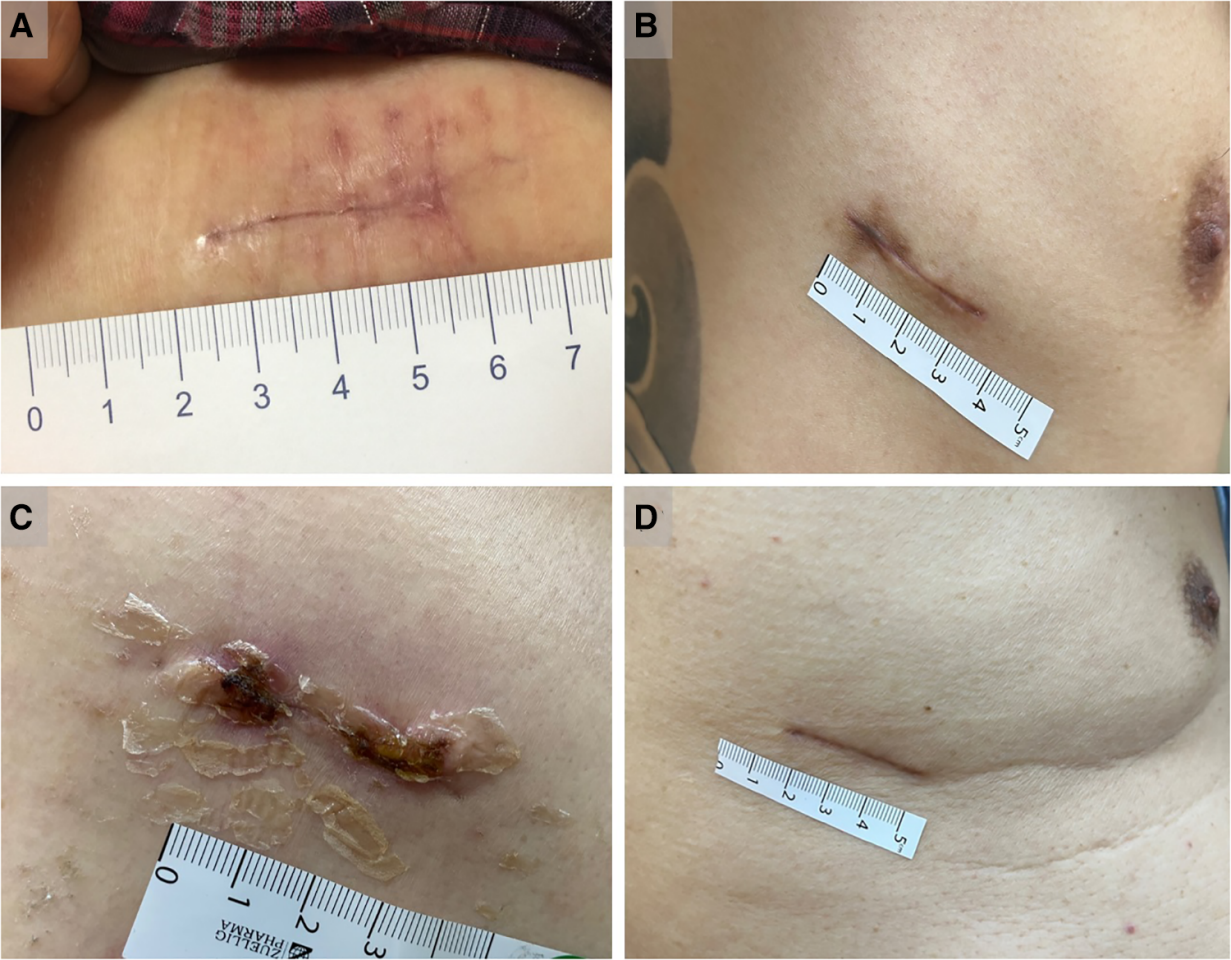
Assessment of scars at the outpatient clinic one month after surgery. (**A**) The representative case with wound scar in group A. (**B**) The representative case with wound scar in group B. (**C**) The representative case with partially peeled off skin adhesives in group C approximately 2 weeks after surgery. (**D**) The representative case with wound scar in group C.

Complications related to chest drain wounds were compared between the conventional and knotless suture groups. Complications included pneumothorax or subcutaneous emphysema after chest tube removal, wound dehiscence or infection, chest tube dislodgement, and incisional effusion. Post-removal pneumothorax was defined as the development of pneumothorax after removal of the chest drain; post-removal subcutaneous emphysema was defined as exacerbation after drain removal; wound dehiscence was defined as the margins of the wound being 2–3 mm or more apart and requiring resewing or conservative treatment; and wound infections such as tenderness, swelling, erythema, purulence, and/or fever. The Patient and Observer Scar Assessment Scale (POSAS) was used by a specially trained physician to assess scars at the outpatient clinic one month after surgery (Figure 4). The POSAS includes objective and subjective assessments and includes six characteristics: pain, itching, color, stiffness, thickness, and irregularity. Six points represented normal skin, and 60 points corresponded to the most severe scar. The complication rate was measured by reviewing all electronic records for each patient's hospitalization period and outpatient clinic follow-ups.

### Statistical analysis

Categorical variables are expressed as numbers with percentages and compared using the chi-square test. Continuous variables were described as means with standard deviations or medians with interquartile ranges (IQR) using analysis of variance (ANOVA) or the Kruskal-Wallis test to compare differences between groups. A two-sided *P*-value <0.05 was considered significant. All data were analyzed using SPSS (version 19.0; SPSS, Chicago, IL, USA).

## Results

**Figure 5:**
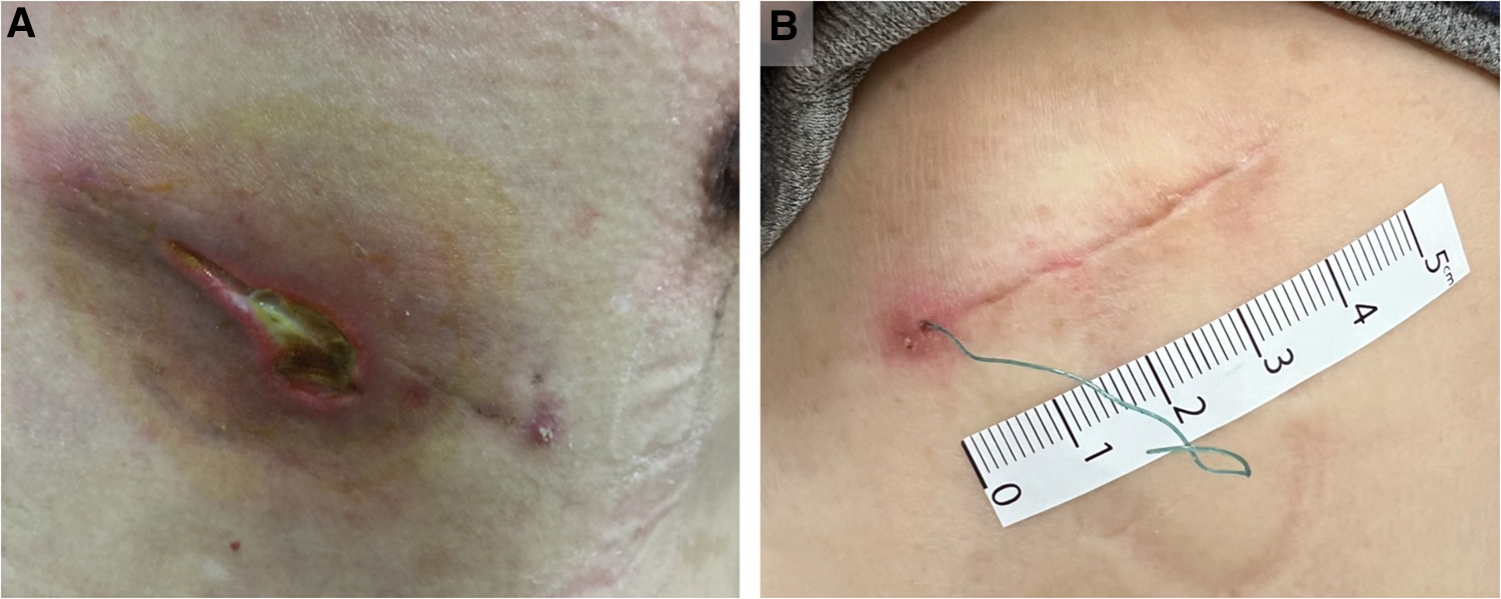
Poor wound healing of representative cases. (**A**) In group B, the representative case with excessive incisional effusion leakage and wound dehiscence. (**B**) In group C, the representative case with mild wound abscess due to an extruded spitting barbed suture.

A total of 334 patients with lung neoplasms who underwent uniportal VATS resection were enrolled over a 5-year period (2016–2021). They were classified into groups A (*n* = 35), B (*n* = 122), and C (*n* = 177) based on different CWC techniques. The clinical characteristics of the patients are summarized in Table 1. There were no significant differences in age, sex, body mass index, smoking status, Charlson Comorbidity Index scores, or incidence of diabetes mellitus among the three groups. Regarding surgical type and pathology, wedge resection was performed more frequently than anatomical resection and primary lung cancer is the predominant histopathology in the three groups. In terms of perioperative data, the median operation time was 60 min in group C, which was lower than that in the other two groups (groups A and B: 70 min) (*p *<* *0.01). In addition, group C also had a shorter median postoperative hospital stay (3 days) than the other two groups (both group A and B:4 days) (*p *< 0.01), as well as shorter median chest tube days (2 days) compared to the other two groups (both group A and B:3 days) (*p *< 0.01). Regarding chest drain-related complications, the incidences of post-removal pneumothorax, post-removal subcutaneous emphysema, post-removal incisional effusion leakage, and wound dehiscence or infection were similar (Table 2). Tube dislodgement was not observed in either group. Only one patient in group B required readmission for local wound debridement because of excessive incisional effusion leakage and wound dehiscence (Figure 5A). Mild wound abscess due to an extruded spitting barbed suture was observed in one patient (Figure 5B). Although statistically insignificant, the percentage of the aforementioned variables was numerically lower in group C than in groups A and B. Notably, in group C, our improved CWC method was superior to the conventional method (group A) with regard to the POSAS scar scale score (*p < 0.01*). However, the differences were not significant between groups B and C after Bonferroni correction.

**Table 1:** Patient characteristics and perioperative data.

	Group A (*n* = 35)	Group B (*n* = 122)	Group C (*n* = 177)	*p* value
Age (mean ± SD) (year)	55.6 ± 18.5	58.7 ± 13.8	57.5 ± 15.2	0.53
Sex (male), *n* (%)	19 (54.2%)	53 (43.4%)	70 (39.5%)	0.26
BMI (mean ± SD) (kg/m^2^)	23.9 ± 2.6	23.5 ± 2.8	24.1 ± 2.2	0.11
Smoking (yes), *n* (%)	5 (14.3%)	16 (13.1%)	21 (11.8%)	0.86
Charlson comorbidity index	2.1 ± 1.6	1.7 ± 1.2	1.8 ± 1.5	0.14
Diabetes mellitus (yes), *n* (%)	6 (17.1%)	25 (20.5%)	33 (18.6%)	0.91
Surgical type				0.87
Wedge resection	28 (80.0%)	95 (77.9%)	135 (76.3%)	
Anatomical resection (segmentectomy/lobectomy)	7 (20.0%)	27 (22.1%)	42 (23.7%)	
Pathology, *n* (%)				0.59
Primary lung cancer	24 (69%)	83 (68%)	133 (75%)	
Secondary lung cancer (metastasis)	3 (8%)	15 (12%)	18 (10%)	
Benign nodule	8 (23%)	24 (20%)	26 (15%)	
Operative time (median with IQR) (min)	70 (60-100)	70 (70–100)	60 (60–90)	<0.01
Blood loss (median with IQR) (ml)	10 (10–20)	10 (10–20)	20 (15–20)	0.08
Hospital days (median with IQR) (d)	4 (3–5)	4 (3–4)	3 (3–4)	<0.01
Chest tube days (median with IQR) (d)	3 (2–4)	3 (2–3)	2 (1–3)	<0.01

SD, standard deviation; BMI, body mass index; IQR: interquartile range.

**Table 2:** Chest drain-related complications and wound scarring.

	Group A (*n* = 35)	Group B (*n* = 122)	Group C (*n* = 177)	*p*-value
Chest drain-related complication, *n* (%)	7 (20%)	28 (23%)	28 (15.8%)	0.61
Post-removal pneumothorax	2 (5.7%)	6 (5%)	8 (4.5%)	
Post-removal subcutaneous emphysema	3 (8.5%)	11 (9%)	13 (7.3%)	
Post-removal incisional effusion leakage	1 (2.9%)	7 (5.7%)	3 (1.7%)	
Wound dehiscence/infection/inflammation	1 (2.9%)	4 (3.3%)	4 (2.3%)	
Tube dislodgement	0	0	0	
POSAS scar scale	9.5 ± 4.3	7.6 ± 2.5	7.2 ± 2.1	<0.01

POSAS, Patient and Observer Scar Assessment Scale. In the POSAS scar scale, the lower scores, the better the scar status.

## Discussion

Uniportal VATS was first introduced by Dr. Rocco in 2004 for its advantages of minimally invasive surgery (9), and later, Dr. Gonzale-Rivas improved it by performing complex thoracoscopic lobectomy through a single incision (10). Until now, uniportal VATS has been progressively gaining popularity among thoracic surgeons, and its application has expanded to various types of thoracic procedures (11–13). After thoracic surgery, placement of a chest tube is often necessary to drain excessive intrathoracic fluid or air during postoperative care. Chest tube-related complications, including pneumothorax, subcutaneous emphysema, tube dislodgement, poor wound healing, and infection, can occur due to improper tube fixation and inadequate closure of the thoracotomy or VATS incision (14). In the case of uniportal VATS, the direct insertion of the chest tube through a surgical incision makes conventional CWC methods insufficient to effectively reduce the leakage of fluid or air into or out of the intrathoracic space (5). This inadequate closure may not minimize the risk of complications such as pneumothorax, wound infection, subcutaneous emphysema, and the development of unsightly wound healing scars after uniportal VATS (15). Furthermore, the majority of conventional CWC methods, such as the purse-string technique, skin stapler, interrupted vertical mattress sutures, and non-absorbable suture tube anchoring, require their removal after the extraction of the chest tube. This additional step places an extra burden on the medical staff and causes considerable discomfort to patients (4). In group A, we used a skin stapler and a 3-0 Nylon tube anchored by petrolatum gauze as the conventional CWC method. As expected, significantly longer operative times, postoperative hospital days, chest tube days, and worse scar conditions were observed when compared with our modified CWC method (groups B and C). Although not statistically significant, higher chest tube-related complications were observed in the conventional group. Therefore, thoracic surgeons worldwide have attempted to optimize the CWC method for uniportal VATS.

In 2015, Son et al. proposed a new technique for creating a separate chest tube insertion through another incision below the intercostal port, which was then closed and anchored using Vicryl and Nylon sutures (5). Direct tube insertion issues in uniportal VATS can be prevented, and no wound-related complaints have been reported; however, the stitches still need to be removed (5). Subsequently, several similar modified chest tube wound closure methods were proposed (4, 6, 7, 16–19). The removal of stitches with a firm wound closing effect due to its innovative suture material, which is the absorbable barbed thread, was first introduced by Kim et al. for VATS incisions in 2017 (19); later in 2018, they showed non-inferiority in complication rates compared to the conventional method (16). Xu et al. then utilized it in 50 patients undergoing uniportal VATS to strengthen the muscle and intradermal closure and fix the tube without the need for suture removal; however, they still reported two cases of postoperative subcutaneous emphysema (4). Haitao Xu et al. then introduced their “shingled suture” including intermittent Vicryl suture for deep muscle and subcutaneous layer and removal-free continuous barbed suture only for intradermal layer with 1-0 Ethicon for tube fixation in multiportal VATS settings (18). Superiority in post-removal complications and better scar conditions were shown in their modified method, but it requires multiple steps that are more time-consuming than the conventional method (18). In 2022, Chen et al. retrospectively enrolled 258 patients who underwent uniportal VATS and compared their modified full-layer barbed suture technique with the conventional interrupted method (7). Only the incidence of subcutaneous emphysema after tube removal was significantly reduced with their modified technique (7), and the evaluation of wound healing or scar conditions was lacking in their study. The relevant literature is summarized in Table 3. Notably, our study has several strengths. First, our study may be one of the largest case series to describe and compare three different types of CWC methods including conventional (group A), modified (group B), and ameliorated versions (group C) following uniportal VATS lung resections. Second, all procedures were performed by the same team of thoracic surgeons; all chest tubes were removed using the same technique, and all patients had similar chest tube management. Thus, interpersonal or institutional biases may be largely eliminated. Third, in our ameliorated CWC technique (group C), we used Vicryl for muscle and subcutaneous closure and barbed V-loc for intradermal closure, followed by topical skin adhesive covering. After pulling out the pre-embedded barbed suture end for wound closure during the tube removal, skin adhesive was immediately applied over the wound to optimize the sealing effect. Topical skin adhesives are believed to be the optimal deep dermal wound closure tools capable of maintaining high tension in the wound conjunction to promote healing (20). Thus, accidental loosening of the barbed suture is reduced. All complications in the three groups were mild and mitigated without the need for medical intervention, except for one patient in group B who required surgical reintervention for wound dehiscence. Our modified CWC method in uniportal VATS is comparable to the relevant literature in terms of safety and incidence of chest tube-related complications. Furthermore, our removal-free barbed (knotless) suture plus topical skin adhesive application (group C) may not only lower the clinician's workload, but also satisfy the patients’ wound cosmesis and reduce the burden of postoperative wound care.

**Table 3:** Published studies on chest tube-related complications using the new methods.

Author	Publication year	Number of patients receiving new method	Chest tube size	Barbed suture material (Brand name)	Post-removal pneumothorax	Post-removal subcutaneous emphysema	Post-removal incisional effusion leakage	Wound dehiscence/infection	Tube dislodgement	Scar condition
Kim MS et al. (16)	2018	85	NA	Stratafix or V-loc	2.3% (2/85)	NA	NA	1/85 (1.2%)	No	NA
Fu R et al. (17)	2019	71	20Fr	No	NA	NA	8.5% (6/71)	4.2% (3/71)	1.4% (1/71)	Superior to conventional group
Xu Y et al. (4)	2019	50	24Fr	Stratafix	NA	4% (2/50)	NA	NA	No	NA
Xu H et al. (18)	2021	67	NA	Angiotech	0	NA	1.5% (1/67)	0	No	Superior to conventional group
Chen Z et al. (7)	2022	131	28Fr	QUILL	1.5% (2/131)	2.3% (3/131)	1.5% (2/131)	3.1% (4/131)	No	NA
Shi W et al. (6)	2023	72	20Fr	No	NA	NA	2.8% (2/72)	2.8% (2/72)	No	Superior to conventional group
Current study		177	16 or 20Fr	V-loc	4.5% (8/177)	7.3% (13/177)	1.7% (3/177)	2.3% (4/177)	No	Superior to conventional group

NA, not available; Fr, French size.

The main limitations of this study were its retrospective design without randomization of patients, lack of consideration of the factors involved in wound healing, and documentation of the duration for each suture. Another concern is cost, as the knotless suture material and topical skin adhesives are certainly costlier than those in the conventional suture group. This issue should be addressed in a cost-benefit framework, despite the fact that this ameliorated method is worthy of use because of the patient's high level of satisfaction.

In conclusion, our improved CWC technique is as effective as the conventional approach while improving the esthetic appearance of the wound. Utilizing a continuous suture with a removal-free barbed suture along with the appropriate application of topical skin adhesives may be a favorable CWC method for uniportal VATS.

## Data Availability

The raw data supporting the conclusions of this article will be made available by the authors, without undue reservation.

## Supplementary material

Supplementary Video S1 Utility of Vaseline® Petrolatum gauze to wound after chest drain removal.

Supplementary Video S2 Barbed suture for chest drain wound closure in uniportal VATS.

Supplementary Video S3 Utility of topical skin adhesive to wound after chest drain removal.
